# Exploring associations of maternal exposure to ambient temperature with duration of gestation and birth weight: a prospective study

**DOI:** 10.1186/s12884-018-2100-y

**Published:** 2018-12-29

**Authors:** Shenghui Li, Jiajia Wang, Zhiwei Xu, Xiaoyu Wang, Gang Xu, Jun Zhang, Xiaoming Shen, Shilu Tong

**Affiliations:** 1 School of Public Health, Shanghai Jiao Tong University, Shanghai, China; 2 MOE - Shanghai Key Laboratory of Children’s Environmental Health, Xinhua Hospital, School of Medicine, Shanghai Jiao Tong University, Shanghai, China; 3 School of Public Health and Social Work, Institute of Health and Biomedical Innovation (IHBI), Queensland University of Technology, Brisbane, Australia

**Keywords:** Duration of gestation, Birth weight, Temperature, Climate change

## Abstract

**Background:**

Evidence suggests the possible impact of ambient high temperature on fetal growth and birth outcomes. However, little is known about the relative impact of exposure to heat and cold and the possible vulnerable window during pregnancy.

**Methods:**

Data on a total of 237,585 pregnant women from January 1st, 2001 to December 31st, 2010 were acquired from the Queensland Health, Australia. Daily data on meteorological factors, including ambient temperature, relative humidity, barometric pressure, and air pollutants, such as PM_10_, SO_2_, NO_2_, and O_3_, were obtained from relevant government agencies. This study was to examine the associations of maternal exposure to ambient temperature (high and low temperatures, in early vs. late pregnancy) with the duration of gestation and birth weight.

**Results:**

A J-shaped association between minimum temperature at conception and duration of gestation was observed after adjusting for seasonality and other confounders. Compared to women who were exposed to the minimum temperature of 15–20 °C in the first gestational week, exposure to the minimum temperature of > 20 °C significantly increased the duration of gestation by 0.029 weeks (95% CI: 0.008, 0.049). A cumulative effect was found when exposure across the first four weeks was examined. There was an inverted U-shaped relationship between minimum temperature at delivery and the duration of gestation. Compared to women exposed to 15–20 °C, exposure to minimum temperature of > 20 °C and ≤ 10 °C was associated with a shortened gestation by 0.030 weeks (95% CI: -0.052, − 0.008) and 0.018 weeks (95% CI: -0.057, − 0.004), respectively. By contrast, an inverse relationship between maximum temperature and birth weight was observed. Compared to exposure to the maximum temperature of > 30 °C in the last week of pregnancy, maternal exposure to 20–25 °C and < 20 °C significantly increased birth weight by 0.011 kg (95% CI: 0.008, 0.018) and 0.018 kg (95% CI: 0.010, 0.031), respectively. Similarly, a mild cumulative effect was observed when maximum temperature exposure across the four weeks before delivery was evaluated.

**Conclusions:**

The finding emphasized the importance of keeping an optimal temperature range during pregnancy for reducing the risk of preterm birth and low birthweight.

**Electronic supplementary material:**

The online version of this article (10.1186/s12884-018-2100-y) contains supplementary material, which is available to authorized users.

## Background

Climate has frequently been changing throughout the Earth’s history. However, the recent pace of warming far exceeds that of any previous warming episode in the past 10,000 years [1]. From 1880 to 2012, the global surface average temperature has increased by 0.85 °C and the largest increase has occurred after the 1970s [2, 3]. Global climate change is anticipated to have multiple impacts on human health, many of them adverse and some severe, but most of these impacts remain to be quantified [4, 5]. Pregnant women are particularly sensitive to weather conditions and environmental exposure due to their hormone-related physiological changes, physical agility, and variation in immunity and mood [6–8].

Previous studies have explored the short-term and long-term effects of maternal exposure to heat stress on preterm birth, stillbirth, and birth weight [9–17]. In general, existing evidence seems to support an association between high temperature exposure and adverse pregnancy outcomes [9–17]. If the association between high temperature and birth outcomes is valid, the effects of exposure to cold conditions should also be assessed. However, only a few studies have explored the possible impact of cold temperature on fetal development and the results are inconsistent [14, 16, 18, 19]. For example, a retrospective analysis of 3333 singleton live births for more than 36 weeks of pregnancy in Turkey found a relationship between cold ambient temperature and low birth weight [18]. However, another similar study in New Zealand did not find a significant relationship [19].

The effect of ambient temperature on birth outcomes may depend on the stage of gestation, with a possible susceptible period during pregnancy [20, 21]. For example, a study among 147,357 singleton live births in Perth, Western Australia, from 1998 to 2006, found a 9.15 °C increase in ambient maximum temperature during the third trimester was associated with a decrease in proportion of optimal birth weight by 0.14%. In the first and second trimesters, however, the impact was not found [21]. The identification of a possible vulnerable exposure window has important clinical and public health implications. However, little research has been conducted on this issue.

Based on above, we speculated the possibility that high temperature and cold condition could have varied health effect, and, meanwhile, the effect may depended on different exposure period of gestation. This study focused on two important birth outcomes, duration of gestation and birth weight, aimed to address two issues: 1) what is the overall impact of both hot and cold temperatures on duration of gestation and birth weight? 2) is there any possible susceptible period to temperature exposure for different pregnant outcomes?

## Methods

### Study subjects

Participants in this study were all the singleton births born in Brisbane, Australia, between January 1st, 2001 and December 31st, 2010. Data were collected from the Perinatal Data Collection Unit (PDCU) of the Queensland Health Statistics Centre. All births were recorded according to the following criteria: 1) at least 20 weeks of gestation; or 2) at least 400 g in birth weight; 3) born in Queensland including all public hospitals, private hospitals, and private midwifery and medical practices.

**Fig. 1:**
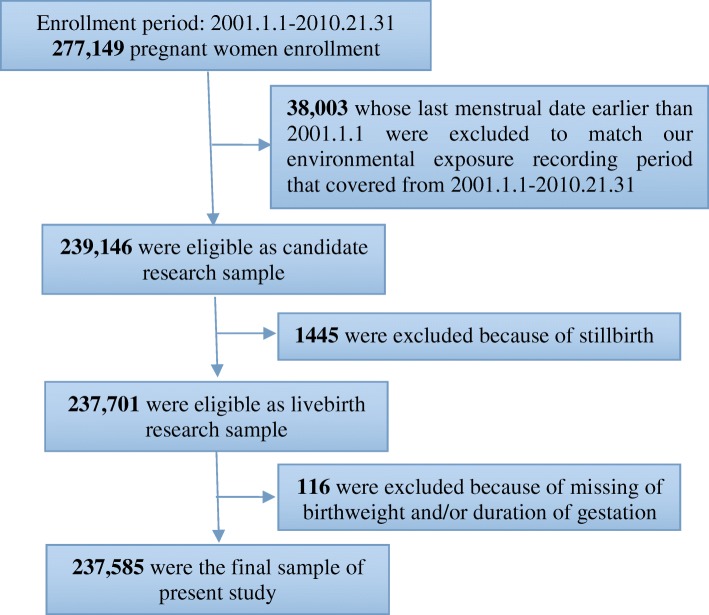
Study Flowchart and Participants Enrollment. The study flowchart and participants enrollment of singleton births

In this study only live birth was eligible. Among all live births, those whose last menstrual date was earlier than January 1st 2001 were excluded to match our environmental exposure records that covered from January 1st 2001 to December 31st 2010. The final sample consisted of 237,585 singleton live births. The study profile and participants selection process are shown in Fig. 1.

### Definition of maternal and perinatal characteristics

The following variables were collected based on standardized maternal medical records: duration of gestation, date of last menstrual period, date of birth, gender of baby, birth weight, mode of labor onset (spontaneous, induced, and caesarean), maternal age group, marital status (Married/cohabiting, Divorced/separated, and Never married), Indigenous status, and parity.

### Meteorological factors

Meteorological factors, including daily maximum and minimum temperatures, relative humidity at 9:00 am and 15:00 pm, and ambient barometric pressure at 9:00 am and 15:00 pm, were recorded in nine monitoring stations in Brisbane during the study period.

### Air pollutants

Air pollutants, including particulate matter with a diameter < 10 μm (PM10), ozone (O_3_), nitrogen dioxide (NO_2_), and sulfur dioxide (SO_2_), were recorded in five monitoring stations in Brisbane.

### Statistical analysis

We calculated the weekly mean maximum temperature, minimum temperature, relative humidity, and ambient barometric pressure, as well as weekly pollutant levels (PM_10,_ O_3,_ NO_2,_ and SO_2_) based on the original daily data. Since the date of conception and birth varied among different pregnant women, we, then, fitted weekly data of these environment indicators to each pregnant woman, using their mean levels in the first/last four weeks of the corresponding gestation. To compare the possible acute and cumulative effects of temperature exposure, the exposures in the first one gestational week vs. in the first four gestational weeks and in the last gestational week vs. in the last four gestational weeks were examined, respectively, in the subsequent analyses. Descriptive statistics for meteorological and pollutant indicators at conception/delivery are shown in (Additional file 1: Table S1).

Summary statistics and distributional plots were checked for all variables. Statistical descriptions were made by use of the mean, standard deviation for continuous variables, and percentage for categorical variables. One-way ANOVA was used to compare differences between groups.

The potential nonlinear association of ambient temperature with duration of gestation/birth weight was examined using penalized splines in generalized additive models (GAM). Generalized cross validation was used to automatically select the degree of smoothing for spines. Based on the distribution of temperature and overall effect of temperature on duration of gestation/birth weight in GAM, maximum temperature was recorded into following four groups as > 30 °C, 25–30 °C, 20–25 °C, and ≤ 20 °C, while minimum temperature was grouped as > 20 °C, 15–20 °C, 10–15 °C, and ≤ 10 °C.

Generalized linear regression models were further applied to estimate the crude and adjusted associations between maximum/minimum temperature groups (X, independent variables) and duration of gestation/birth weight (Y, dependent variables). Adjustments were made following a three-step procedure. Model 1 was adjusted for all maternal and perinatal variables (as listed in Table 1). In model 2, relative humidity and air pressure, along with all air pollutants (PM_10,_ O_3,_ NO_2,_ and SO_2_) around conception and delivery, were further controlled. Accumulating studies documented that other seasonal factors (e.g., nutrition and physical activity), independent of ambient temperature, may affect fetal development and birth outcomes [2, 10, 22–24]. Therefore, we controlled for seasonal confounding by including a calendar month of delivery as a dummy variable in the final model. Moreover, temperature around delivery were simultaneously taken into account when examining the health effect of temperature exposure at conception (Model 3^a^), and temperature around conception were simultaneously taken into account when examining the health effect of temperature exposure at delivery (Model 3^b^).

**Table 1:** The summary statistics of duration of gestation and birthweight by maternal/perinatal characteristic in Brisbane, 2000-2010 (n=237,585)

Variable (No, %)	Duration of gestation, weeks		Birth weight, kg	
	Mean	SD	Mean	SD
Maternal characteristics				
Age at delivery				
<20 (12775, 5.38)	39.06	2.10	3.327	0.578
20-35 (178029, 74.93)	38.99	1.85	3.429	0.556
≥35 (46781, 19.69)	38.88	1.88	3.414	0.569
*F*/*p value*	*385.95/<.001*		*198.75/<.001*	
Indigenous status				
Indigenous (4650, 1.96)	38.68	2.32	3.295	0.626
Non-indigenous (232935, 98.04)	38.94	1.86	3.423	0.559
*F*/*p value*	*92.07/<.001*		*237.62/<.001*	
Marriage				
Married/cohabiting (205243, 86.39)	38.94	1.83	3.432	0.554
Divorced/separated (28737, 12.10)	38.96	2.13	3.343	0.593
Never married (3605, 1.52)	38.83	1.93	3.367	0.577
*F*/*p value*	*8.75/<.001*		*335.33/<.001*	
Parity				
Primiparity (99742, 41.98)	39.09	1.96	3.369	0.560
Multiparity (137843, 58.02)	38.85	1.79	3.457	0.558
*F*/*p value*	*783.07/<.001*		*1438.93/<.001*	
Perinatal characteristics				
Gender				
Male (122374, 51.51)	38.91	1.92	3.480	0.572
Female (115211, 48.49)	38.97	1.82	3.358	0.541
*F*/*p value*	*54.67/<.001*		*2848.04/<.001*	
Mode of laboronset				
Spontaneous (132904, 55.94)	39.02	1.89	3.408	0.545
Induced (55341, 23.29)	39.50	1.62	3.523	0.537
Caesarean (49340, 20.77)	38.06	1.78	3.338	0.608
*F*/*p value*	*8651.48/<.001*		*1501.11/<.001*	
Duration of gestation at birth, wk				
≥39 (160888, 67.72)	39.85	0.77	3.574	0.449
37-39 (61650, 25.95)	37.77	0.42	3.272	0.461
34-37 (11097, 4.67)	35.36	0.76	2.675	0.472
<34 (3950, 1.66)	29.98	2.38	1.554	0.614
*F*/*p value*	*40154.50/<.001*		*40154.50/<.001*	
Birth weight for gestation				
SGA (23161, 9.75)	38.53	2.46	2.654	0.408
AGA (214424, 90.25)	38.98	1.79	3.503	0.510
*F*/*p value*	*1225.05/<.001*		*60148.60/<.001*	

Abbreviations: *SGA* small for gestational age, *AGA* appropriate for duration of gestation

All analyses were performed using the Statistical Analysis System (SAS) for Windows, version 9.2 (SAS Institute, Cary. NC) and R version 2.15.1 (The R Foundation for Statistical Computer, www.r-project.org). In the presentation of the results, the statistical significance was set at *p* value < 0.05 (two tailed).

## Results

### Duration of gestation and birth weight stratified by maternal/perinatal characteristics

The present study included 237,585 subjects. Of these, 5.38, 74.93, and 19.69% were < 20, 20–35, and ≥ 35 years at delivery, respectively. The mean duration of gestation at birth was 38.94 weeks (SD = 1.87, ranged from 20 weeks to 43 weeks), and the mean birth weight was 3.42 kg (SD = 0.56, ranged from 0.40 to 6.7 kg). Table 1 shows the maternal/perinatal characteristics of the study sample.

### The associations of ambient temperature exposure with duration of gestation/birth weight

#### Exploratory analysis

**Fig. 2:**
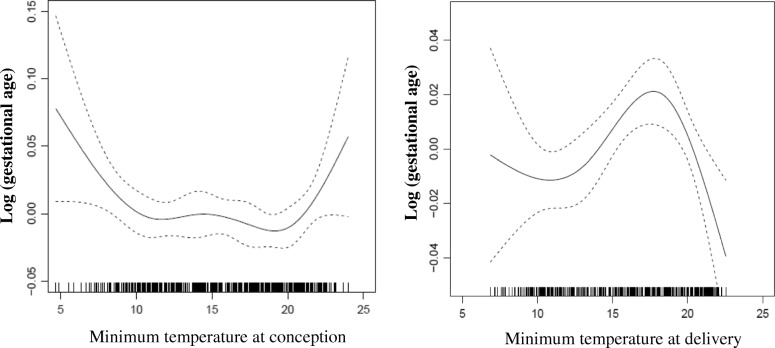
Minimum Temperature and Gestational Age

The association between ambient temperature and duration of gestation/birth weight was explored by penalized splines in generalized additive models. We observed a J-shaped association of duration of gestation with both minimum temperatures at conception and at delivery (both *p <* 0.001). The smoothed plots of nonlinear associations of duration of gestation with minimum temperature at the first/last gestational week are shown in Fig. 2. The very similar nonlinear associations of duration of gestation with minimum temperature at the first/last four gestational weeks were also identified (Additional file 2: Figure S1**,** both *p <* 0.001**)**. By contrast, approximate linear trend or wave curves, without clear correlates, were observed between maximum temperature, either at concept or at delivery, with duration of gestation (Additional file 3: Figure S2 and Additional file 4: Figure S3**,** all *p >* 0.05).

**Fig. 3:**
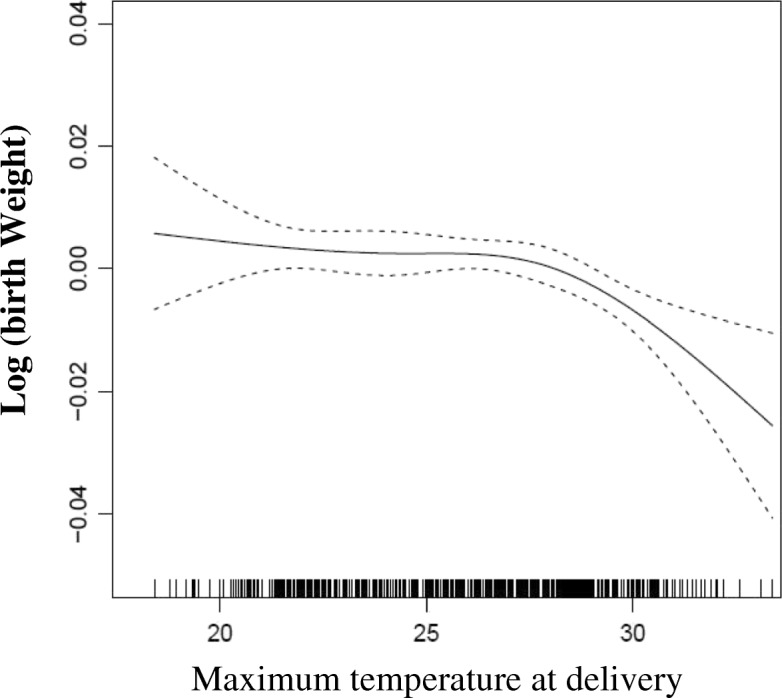
Maximum Temperature and Gestational Age

The smoothed plots of association between birth weight and maximum temperature at the last gestational week is shown in Fig. 3, which indicates a approximate linear association (similar plots was observed between birth weight and maximum temperature at the last four weeks of pregnancy, Additional file 5: Figure S4, both *p <* 0.05**)**. Associations of birth weight with maximum temperature at concept or minimum temperature at concept/delivery were also explored. Generally, there were wavy curves without clear correlates (not shown).

#### Comparative analysis of duration of gestation/birth weight across different temperature categories

Table 2 reveals the results of duration of gestation/birth weight across different ambient temperature categories. The duration of gestation statistically varied between different minimum temperature groups at both concept and delivery. By contrast, birth weight shows statistically significant differences between temperature groups (at both conception and delivery, either maximum or minimum temperature).

**Table 2.** The description of duration of gestation and birth weight by different ambient temperature category in Brisbane, 2000-2010 (n=237,585)

	Duration of gestation, weeks		Birth weight, kg	
	Mean	SD	Mean	SD
In the first week				
Maximum temperature				
>30	38.93	1.87	3.423	0.561
25-30	38.94	1.84	3.424	0.558
20-25	38.94	1.91	3.414	0.564
≤20	38.96	1.85	3.424	0.555
*F*/*p value*	1.28/0.298		*6.31/<.001*	
Minimum temperature				
>20	38.95	1.82	3.430	0.555
15-20	38.92	1.86	3.420	0.560
10-15	38.93	1.90	3.414	0.563
≤10	38.96	1.92	3.420	0.565
*F*/*p value*	*3.78/0.010*		*7.78/<.001*	
In the first four weeks				
Maximum temperature				
>30	38.92	1.83	3.425	0.558
25-30	38.94	1.85	3.423	0.558
20-25	38.94	1.92	3.415	0.565
≤20	38.98	1.80	3.425	0.540
*F*/*p value*	0.72/0.539		*3.85/0.009*	
Minimum temperature				
>20	38.96	1.82	3.431	0.556
15-20	38.92	1.85	3.419	0.558
10-15	38.92	1.92	3.417	0.566
≤10	38.98	1.87	3.421	0.558
*F*/*p value*	*9.47/<.001*		*10.53/<.001*	
In the last week				
Maximum temperature				
>30	38.93	1.89	3.407	0.565
25-30	38.95	1.88	3.421	0.561
20-25	38.93	1.85	3.422	0.558
≤20	38.93	1.88	3.431	0.563
*F*/*p value*	1.23/0.298		*6.31/<.001*	
Minimum temperature				
>20	38.92	1.91	3.411	0.564
15-20	38.95	1.88	3.422	0.562
10-15	38.94	1.84	3.423	0.557
≤10	38.93	1.85	3.426	0.559
*F*/*p value*	*3.68/0.0112*		*6.28/<.001*	
In the last four weeks				
Maximum temperature				
>30	38.95	1.87	3.411	0.562
25-30	38.94	1.89	3.419	0.561
20-25	38.93	1.84	3.425	0.559
≤20	38.92	1.99	3.425	0.566
*F*/*p value*	0.84/0.472		*4.58/0.003*	
Minimum temperature				
>20	38.93	1.89	3.407	0.562
15-20	38.95	1.88	3.417	0.560
10-15	38.93	1.85	3.424	0.559
≤10	38.93	1.86	3.424	0.563
*F*/*p value*	*3.38/0.017*		*6.78/<.001*	

#### Crude and adjusted associations of ambient temperature with duration of gestation

Table 3 shows that minimum temperature at both conception and delivery was associated with duration of gestation. In the unadjusted model, compared to those exposed to a minimum temperature of 15–20 °C in the first week of pregnancy, > 20 °C and ≤ 10 °C could significantly lengthen the duration of gestation. A cumulative effect was found when exposure across the first four weeks was examined. Through three-step controlling, this association partly changed. In the final full model (Model 3), minimum temperature ≤ 10 °C was not associated with the duration of gestation anymore, meanwhile, > 20 °C either in the first week or in the first four weeks retained to be significantly related to the increase of duration of gestation by 0.029 weeks (*p* = 0.006) and 0.049 weeks (*p <* 0.001).

**Table 3:** Association of ambient temperature at conception/delivery with duration of gestation in Brisbane, 2000-2010 (n=237,585)

	Duration of gestation, weeks							
	Crude		Adjusted					
			Model 1		Model 2		Model 3^a/b^	
	β (95% CI)	*P value*	β (95% CI)	*P value*	β (95% CI)	*P value*	β (95% CI)	*P value*
In the first week								
Maximum temperature								
>30	Ref		Ref		Ref		Ref	
25-30	0.008 (-0.017, 0.033)	0.539	0.003 (-0.016, 0.021)	0.791	-0.005 (-0.025, 0.015)	0.598	0.000 (-0.020. 0.021)	0.982^a^
20-25	0.007 (-0.018, 0.033)	0.572	0.023 (0.004, 0.043)	*0.021*	-0.006 (-0.034, 0.021)	0.664	0.009 (-0.021, 0.038)	0.571^a^
≤20	0.026 (-0.026, 0.079)	0.322	0.031 (-0.009, 0.070)	0.135	-0.004 (-0.050, 0.041)	0.854	0.006 (-0.041, 0.052)	0.814^a^
Minimum temperature								
>20	0.026 (0.005, 0.046)	*0.013*	0.007 (-0.008, 0.023)	0.371	0.022 (0.003, 0.040)	*0.020*	0.029 (0.008, 0.049)	*0.006* ^*a*^
15-20	Ref		Ref		Ref		Ref	
10-15	0.004 (-0.015, 0.023)	0.684	0.015 (0.001, 0.029)	*0.040*	-0.013 (-0.031, 0.007)	0.198	-0.018 (-0.038, 0.002)	0.082^a^
≤10	0.033 (0.008, 0.058)	*0.009*	0.033 (0.014, 0.051)	*<.001*	-0.004 (-0.029, 0.022)	0.771	-0.010 (-0.038, 0.018)	0.481^a^
In the first four weeks								
Maximum temperature								
>30	Ref		Ref		Ref		Ref	
25-30	0.007 (-0.020, 0.034)	0.593	0.008 (-0.012, 0.029)	0.427	-0.003 (-0.025, 0.020)	0.822	0.013 (-0.011, 0.036)	0.305^a^
20-25	0.014 (-0.014, 0.042)	0.320	0.030 (0.009, 0.051)	*0.006*	-0.010 (-0.042, 0.022)	0.529	0.020 (-0.017, 0.056)	0.288^a^
≤20	0.054 (-0.041, 0.148)	0.265	0.037 (-0.034, 0.108)	0.310	-0.005 (-0.082, 0.072)	0.893	-0.008 (-0.086, 0.069)	0.833^a^
Minimum temperature								
>20	0.035 (0.014, 0.055)	*0.001*	0.015 (-0.001, 0.030)	0.063	0.032 (0.014, 0.051)	*<.001*	0.049 (0.026, 0.072)	*<.001* ^*a*^
15-20	Ref		Ref		Ref		Ref	
10-15	0.007 (-0.012, 0.025)	0.470	0.018 (0.004, 0.032)	*0.012*	-0.010 (-0.031, 0.011)	0.338	-0.025 (-0.048, 0.001)	0.051^a^
≤10	0.062 (0.036, 0.088)	*<.001*	0.056 (0.036, 0.075)	*<.001*	0.017 (-0.015, 0.049)	0.302	-0.003 (-0.039, 0.033)	0.882^a^
In the last week								
Maximum temperature								
>30	Ref		Ref		Ref		Ref	
25-30	0.019 (-0.007, 0.044)	0.148	-0.006 (-0.025, 0.013)	0.516	-0.003 (-0.022, 0.017)	0.796	0.009 (-0.012, 0.030)	0.412^b^
20-25	0.006 (-0.020, 0.032)	0.669	-0.022 (-0.042, -0.002)	*0.029*	-0.013 (-0.042, 0.016)	0.380	0.003 (-0.028, 0.033)	0.866^b^
≤20	0.002 (-0.049, 0.053)	0.934	-0.043 (-0.082, -0.005)	*0.029*	-0.033 (-0.080, 0.013)	0.160	-0.025 (-0.073, 0.023)	0.302^b^
Minimum temperature								
>20	-0.030 (-0.051, -0.009)	*0.004*	-0.010 (-0.025, 0.006)	0.223	-0.019 (-0.035, -0.002)	*0.033*	-0.042 (-0.061, -0.023)	*<.001* ^b^
15-20	Ref		Ref		Ref		Ref	
10-15	-0.025 (-0.044, -0.007)	*0.008*	-0.024 (-0.038, -0.010)	*<.001*	-0.014 (-0.036, 0.008)	0.221	-0.004 (-0.027, 0.019)	0.732^b^
≤10	-0.013 (-0.038, 0.012)	0.321	-0.023 (-0.042, -0.004)	*0.019*	-0.010 (-0.042, 0.022)	0.546	-0.005 (-0.038, 0.029)	0.777^b^
In the last four weeks								
Maximum temperature								
>30	Ref		Ref		Ref		Ref	
25-30	-0.002 (-0.029, 0.025)	0.891	-0.010 (-0.030, 0.011)	0.345	-0.009 (-0.031, 0.012)	0.399	0.002 (-0.022, 0.025)	0.878^b^
20-25	-0.014 (-0.042, 0.014)	0.338	-0.035 (-0.056, -0.014)	*0.001*	-0.038 (-0.070, -0.005)	*0.024*	-0.022 (-0.058, 0.014)	0.224^b^
≤20	-0.030 (-0.125, 0.066)	0.544	-0.038 (-0.110, 0.034)	0.302	-0.044 (-0.122, 0.033)	0.264	-0.031 (-0.110, 0.049)	0.449^b^
Minimum temperature								
>20	-0.022 (-0.042, -0.002)	*0.034*	-0.002 (-0.017, 0.014)	0.815	-0.003 (-0.021, 0.014)	0.715	-0.030 (-0.052, -0.008)	*0.007* ^b^
15-20	Ref		Ref		Ref		Ref	
10-15	-0.028 (-0.046, -0.009)	*0.003*	-0.029 (-0.043, -0.016)	*<.001*	-0.039 (-0.062, -0.016)	*0.001*	-0.024 (-0.050, 0.001)	0.061^b^
≤10	-0.021 (-0.047, -0.006)	*0.008*	-0.029 (-0.050, -0.008)	*0.007*	-0.043 (-0.078, -0.009)	*0.014*	-0.018 (-0.057, -0.004)	*0.041* ^b^

Model 1: adjusted for maternal age at delivery, indigenous status, maternal marital status, mode of labor onset, parity, baby’s gender, and birth weight

Model 2: further adjusted for relative humidity and air pressure around conception and delivery, along with all air pollutants (PM_10,_ O_3,_ NO_2,_ and SO_2_) around conception and delivery

Model 3^a^: model 2 adjustment plus calendar month at delivery, as well as maximum temperature and minimum temperature around delivery

Model 3^b^: model 2 adjustment plus calendar month at delivery, as well as maximum temperature and minimum temperature around conception

Minimum temperature at delivery was also related to the duration of gestation. Compared to minimum temperature exposure of 15–20 °C in the last week, > 20 °C and 10–15 °C could significantly shorten the duration of gestation, respectively. Similarly, a mild cumulative effect was found. During the process of three-step adjustment, the associations generally maintained. In the final full model (Model 3), exposure to ambient temperature > 20 °C in the last gestational week, as well as > 20 °C and ≤ 10 °C in the last four gestational weeks, were associated with a decreased gestation age, when compared to 15–20 °C, and there were 0.042 weeks (*p <* 0.001), 0.030 weeks (*p* = 0.007) and 0.018 weeks (*p* = 0.041) of the shortened duration of gestation, respectively.

No significant association between maximum temperature and the duration of gestation was detected.

To get more understanding with regard to the associations of ambient temperature with duration of gestation, we also dichotomized duration of gestation into < 37 weeks (preterm birth) and ≥ 37 weeks (full-term birth) to examine the relationship between exposure to ambient temperature and risks of preterm birth (Additional file 6: Table S2 and Additional file 7: Table S3).

#### Crude and adjusted associations of ambient temperature with birth weight

Table 4 depicts the crude and adjusted associations between maternal temperature exposure and birth weight. In the unadjusted model, exposure to maximum and minimum temperature, either at conception or at delivery, was significantly associated with birth weight. After adjustment for only maternal and perinatal characteristics, essentially, the associations were not changed. However, after further controlling for air pollutants, relative humidity, and air pressure around conception and delivery, maximum temperature exposure around conception was not statistically significantly associated with birth weight anymore. In addition, although the association for minimum temperature and birth weight still remained statistically significant, its magnitude has been substantially attenuated. Through the third-step adjustment, only maximum temperature before delivery remained significant. Compared to those exposed to > 30 °C of maximum temperature in the last week of pregnancy, 20–25 °C and ≤ 20 °C could significantly increase the birth weight by 0.011 kg (*p* = 0.041) and 0.018 kg (*p* = 0.024), respectively. A slightly cumulative effect was identified.

**Table 4:** Association of ambient temperature at conception/delivery with birth weight in Brisbane, 2000-2010 (n=237,585)

	Birth weight, kg							
	Crude		Adjusted					
			Model 1		Model 2		Model 3^a/b^	
	β (95% CI)	*P value*	β (95% CI)	*P value*	β (95% CI)	*P value*	β (95% CI)	*P value*
In the first week								
Maximum temperature								
>30	0.010 (0.002, 0.017)	*0.016*	0.010 (0.004, 0.016)	*0.001*	0.005 (-0.004, 0.013)	0.265	0.004 (-0.006, 0.013)	0.447^a^
25-30	0.011 (0.006, 0.016)	*<.001*	0.011 (0.007, 0.015)	*<.001*	0.005 (-0001, 0.011)	0.084	0.005 (-0.001, 0.011)	0.130^a^
20-25	Ref		Ref		Ref		Ref	
≤20	0.010 (-0.004, 0.025)	0.165	0.004 (-0.007, 0.015)	0.473	0.004 (-0.010, 0.016)	0.490	0.003 (-0.009, 0.015)	0.606^a^
Minimum temperature								
>20	0.016 (0.009, 0.022)	*<.001*	0.011 (0.006, 0.016)	*<.001*	0.008 (0.001, 0.017)	*0.048*	0.006 (-0.004, 0.016)	0.215^a^
15-20	0.006 (0.001, 0.012)	*0.033*	0.006 (0.002, 0.011)	*0.004*	0.002 (-0.003, 0.008)	0.417	0.002 (-0.005, 0.008)	0.587^a^
10-15	Ref		Ref		Ref		Ref	
≤10	0.005 (-0.003, 0.013)	0.198	-0.001 (-0.007, 0.005)	0.761	0.002 (-0.004, 0.008)	0.550	0.002 (-0.005, 0.008)	0.601^a^
In the first four weeks								
Maximum temperature								
>30	0.010 (0.002, 0.018)	*0.020*	0.012 (0.006, 0.019)	*<.001*	0.004 (-0.006, 0.014)	0.446	0.001 (-0.011, 0.011)	0.964^a^
25-30	0.008 (0.003, 0.013)	*0.002*	0.009 (0.006, 0.013)	*<.001*	0.001 (-0.006, 0.007)	0.947	-0.001 (-0.008, 0.006)	0.826^a^
20-25	Ref		Ref		Ref		Ref	
≤20	0.010 (-0.017, 0.038)	0.472	0.005 (-0.016, 0.027)	0.642	0.005 (-0.016, 0.027)	0.645	0.003 (-0.020, 0.025)	0.822^a^
Minimum temperature								
>20	0.018 (0.012, 0.024)	*<.001*	0.011 (0.006, 0.016)	*<.001*	0.006 (-0.003, 0.015)	0.190	0.001 (-0.011, 0.012)	0.962^a^
15-20	0.006 (0.001, 0.011)	*0.049*	0.007 (0.003, 0.011)	*0.002*	0.001 (-0.005, 0.008)	0.719	-0.001 (-0.008, 0.007)	0.901^a^
10-15	Ref		Ref		Ref		Ref	
≤10	0.008 (-0.001, 0.016)	0.057	-0.004 (-0.010, 0.003)	0.245	0.000 (-0.007, 0.007)	0.989	0.000 (-0.008, 0.008)	0.998^a^
In the last week								
Maximum temperature								
>30	Ref		Ref		Ref		Ref	
25-30	0.015 (0.007, 0.022)	*<.001*	0.010 (0.004, 0.016)	*<.001*	0.008 (0.002, 0.014)	*0.014*	0.006 (0.000, 0.013)	0.066^b^
20-25	0.015 (0.008, 0.023)	*<.001*	0.014 (0.008, 0.020)	*<.001*	0.008 (-0.001, 0.017)	0.078	0.011 (0.008, 0.018)	*0.041* ^b^
≤20	0.025 (0.009, 0.041)	*0.002*	0.025 (0.013, 0.037)	*<.001*	0.016 (0.002, 0.030)	*0.030*	0.018 (0.010, 0.031)	*0.024* ^b^
Minimum temperature								
>20	Ref		Ref		Ref		Ref	
15-20	0.011 (0.005, 0.017)	*<.001*	0.004 (-0.001, 0.008)	0.141	0.002 (-0.003, 0.007)	0.488	-0.001 (-0.006, 0.006)	0.935^b^
10-15	0.012 (0.005, 0.018)	*<.001*	0.009 (0.004, 0.014)	*<.001*	0.004 (-0.005, 0.013)	0.352	0.002 (-0.008, 0.011)	0.723^b^
≤10	0.015 (0.007, 0.024)	*<.001*	0.012 (0.005, 0.018)	*<.001*	0.005 (-0.007, 0.017)	0.396	0.004 (-0.008, 0.016)	0.510^b^
In the last four weeks								
Maximum temperature								
>30	Ref		Ref		Ref		Ref	
25-30	0.008 (-0.001, 0.016)	0.070	0.007 (0.001, 0.013)	*0.038*	0.005 (-0.002, 0.012)	0.145	0.003 (-0.004, 0.011)	0.349^b^
20-25	0.014 (0.006, 0.023)	*<.001*	0.016 (0.010, 0.023)	*<.001*	0.016 (0.006, 0.026)	*0.002*	0.013 (0.002, 0.024)	*0.021* ^b^
≤20	0.004 (-0.024, 0.033)	0.774	0.012 (-0.011, 0.034)	0.411	0.007 (-0.016, 0.031)	0.544	0.008 (-0.016, 0.033)	0.527^b^
Minimum temperature								
>20	Ref		Ref		Ref		Ref	
15-20	0.011 (0.005, 0.017)	*<.001*	0.005 (0.001, 0.010)	*0.033*	0.004 (-0.002, 0.009)	0.177	0.001 (-0.005, 0.008)	0.703^b^
10-15	0.014 (0.007, 0.020)	*<.001*	0.013 (0.008, 0.018)	*<.001*	0.011 (0.001, 0.020)	*0.029*	0.005 (-0.006, 0.016)	0.363^b^
≤10	0.014 (0.005, 0.023)	*0.003*	0.013 (0.006, 0.020)	*<.001*	0.010 (-0.002, 0.023)	0.113	0.003 (-0.012, 0.018)	0.681^b^

Model 1: adjusted for maternal age at delivery, indigenous status, maternal marital status, mode of labor onset, parity, baby’s gender, and duration of gestation

Model 2: further adjusted for relative humidity and air pressure around conception and delivery, along with all air pollutants (PM_10,_ O_3,_ NO_2,_ and SO_2_) around conception and delivery

Model 3^a^: model 2 adjustment plus calendar month at delivery, as well as maximum temperature and minimum temperature around delivery

Model 3^b^: model 2 adjustment plus calendar month at delivery, as well as maximum temperature and minimum temperature around conception

## Discussion

Results of this study indicate a complex spectrum about the association between ambient temperature exposure and birth outcomes. For the first time, we reported that the duration of gestation seemed to be sensitive to maternal exposure to minimum temperature, while birth weight was susceptible to maximum temperature exposure.

### Ambient temperature exposure and duration of gestation

Most previous studies have focused on the relationship between high temperature and birth outcomes [9–17]. However, within the context of climate change, the frequency and intensity of extreme weather events have increased over the last 30 years [2]. Our study demonstrated that, compared to those exposed to 15–20 °C of minimum temperature in the first week of pregnancy, exposure to ambient temperature > 20 °C significantly increased the duration of gestation by 0.029 weeks. For minimum temperature exposure at the last week before delivery, an inverted U-shape was shown, compared to 15–20 °C, > 20 °C and ≤ 10 °C induced the decrease of gestation by 0.030 weeks and 0.018 weeks, respectively. In addition, if minimum temperature exposure across the longer duration of four weeks was evaluated, a cumulative effect was observed. The finding emphasized the importance of warmth at conception and optimal temperature range before delivery for the duration of gestation.

To our knowledge, this is the first study that has investigated the relation between ambient low temperature and duration of gestation. However, two recent studies examined the impact of ambient low temperature on preterm birth. The one in Uppsala, Sweden set up a retrospective birth cohort among almost 14,000 deliveries from 1915 to 1929 and, in which, it was found that extreme cold exposure adversely affected preterm birth [25]. However, the other one in Rome, Italy among 234,945 singleton live births didn’t find such relationship [26]. The inconsistent results could be partly explained by different patterns of temperature exposure and population characteristics. For example, average minimum temperature during winter in Uppsala, Sweden, is lower than − 10 °C, while average minimum temperature in Rome, Italy, is around 5 °C and rarely below 0 °C [25, 26]. In addition, a number of studies have found adverse effects of high ambient temperature exposure, heat event or heat wave, on the duration of gestation and/or preterm birth [9–13, 26–31]. Our results, in essence, support the previous findings but also suggest that, compared to maximum temperature, the duration of gestation might be more susceptible to the variation in minimum temperature.

### Ambient temperature exposure and birth weight

An almost linear relationship between maximum temperature and birth weight was observed. Compared to exposure to higher than 30 °C at the last week of pregnancy, birth weight significantly increased by 11 g and 18 g for those who exposed to 20–25 °C, and < 20 °C, respectively. Similarly, a mild cumulative effect was observed when maximum temperature exposure across the four weeks before delivery was evaluated. By contrast, minimum temperature was not found to be related to birth weight. The findings indicated that the period before delivery could be a susceptible window for fetal weight growth when high ambient temperature exposure exists.

Among the previous studies on the topic of maternal temperature exposure and birth weight, there was a decrease in birth weight associated with increasing temperature at delivery [21, 28, 32]. A study in Greek examined more than a million deliveries between 1999 to 2003, in which a negative correlation was found between mean ambient temperature during the month of delivery and birth weight (*r* = − 0.22, *P* < 0.01) [29]. An ecological study among 140 populations worldwide showed a significant negative correlation (*r* = − 0.59, *P* < 0.01) between heat index and birth weight, where it was found that a one unit increase in heat index was associated with 2.7% decrease in birth weight [32]. Another study investigated the impact of seasonal variation on fetal growth among 147,357 singleton live births [21]. The results revealed that a 9.15 °C increase in ambient maximum temperature across the third trimester predicted a 0.14% decrease in proportion of optimal birthweight [21]. Our results, along with these findings, consistently demonstrated that fetal growth before delivery was vulnerable to high ambient temperature exposure. Since late pregnancy is a key period for fetal growth [33], this finding may have significant clinical and preventive implications for maternal and perinatal health care. Heat shock proteins are molecular chaperones essential for maintaining cellular functions in respond to environmental challenge. Evidence confirmed that heat stress could induce variations in the expression of heat shock proteins [34]. In addition, heat stress can damage antioxidant defense system and lead to more secretion of oxytoxin [35]. Pregnant women are particularly sensitive to environmental change due to their physical and psychological fragile condition [6–8]. The impaired immune defensive function and the higher level of oxidative stress could affect maternal health and fetal growth.

A few studies have explored the possible impact of cold ambient temperature on birth weight [14, 16, 18, 19, 25, 36], but results are inconsistent. Three of them found a reduced birth weight when exposure to cold temperature during mid-pregnancy [18, 36] or in the third trimester [16]. The potential mechanism underlying the association between cold temperature and low birth weight was partly due to the decreased exposure to sunshine which may result in lower levels of vitamin D [19, 37]. Research suggested that vitamin D is essential for normal placental function and, therefore, fetal growth [38]. In addition, cold temperature was found to be associated with a series of changes of peripheral vascular function, including higher blood pressure, peripheral vasoconstriction, increased platelet count, and lower blood viscosity, and tt has been proposed that all thhe changes were associated with increased sympathetic activity and placental dysfunction [39, 40]. Although maternal cold exposure may hinder fetal growth, the present study didn’t find the significant relationship between between cold temperature and birth weight. Study locations need to be taken into account when comparing different studies. Brisbane is a subtropical city that barely experiences extremely cold temperatures. The climate characteristics of our study location may hinder us from exploring the influence of extreme cold stress.

### Strengths and limitations

It is crucial to identify the possible vulnerable exposure window for a specific birth outcome. However, a methodological challenge arises when dealing with preterm birth, when pregnancy doesn’t consist of three full trimesters. The common compromised approach was to focus on exposure during the period before delivery [9–13, 26–30] or, alternatively, to examine temperature exposure by trimester only among full-term birth [16, 18, 36]. The major strength of this study is to fit weekly environment data in the first four weeks and the last four weeks by the date of conception and delivery, respectively. This approach made it possible to examine the association of maternal temperature, both early and late pregnancy, with birth outcomes among all singleton live births, including full-term births and preterm births. Previous studies reported that there is the possibility of a spurious or biased association between temperature exposure and birth outcomes if only a specific period exposure was examined [20, 21]. In reality, exposure to higher temperature before delivery usually intertwines with their exposure to cooler temperature in early pregnancy. Our analyses alleviated this problem to a certain extent. In addition, we used maximum and minimum temperatures instead of mean temperature, making it easier to find the impact of extreme temperature exposure. Moreover, as demonstrated in this study, the different influences of maximum temperature and minimum temperature on birth outcomes were observed.

Several limitations should be acknowledged in interpreting the results. The principal limitation lies in ecological assessment of exposure, both for meteorological indicators and air pollutants. Personal exposure may be modified or attenuated by the duration spent indoors. In addition, the present study relied on the mothers’ report of the date of the last menstrual period to determine gestational age at birth, gestational weeks were used as an analytic scale in this study. A 24 h time frame may be not sufficiently accurate to quantify the difference of several hours, but this bias is likely to be non-differential and to lead the estimates towards null hypothesis. Moreover, as a population-based study, even a small reduction in mean gestational age can lead to a considerable increase in the occurrence of preterm birth. Finally, although we controlled several possible confounding factors, other factors, such as health condition and lifestyle behaviors, may be significantly associated with birth outcomes.

## Conclusion

This study provided new insights and enriched the understanding of the relationship between ambient temperature and birth outcomes through the following findings: (I) There were different pregnancy periods vulnerable to ambient temperature exposure for different birth outcomes. For the duration of gestation, both the early and late pregnant periods were important; while for birth weight, the late pregnant period was more important than the early period. (II) Duration of gestation seemed to be sensitive to minimum temperature, while birth weight was susceptible to maximum temperature. (III) A J-shaped and an inverted U-shaped associations were observed between the duration of gestation and maternal minimum temperature exposure at conception and delivery, respectively. Meanwhile, there appeared to be an almost linear relationship between maternal exposure to maximum temperature and birth weight.

## Additional files

Additional file 1: **Table S1.** Summary distribution of meteorological and pollutant exposure of 237,585 births in Brisbane, 2000–2010. Summary distribution of meteorological and pollutant exposure. (DOCX 27 kb)

Additional file 2: **Figure S1.** The associations between ambient minimum temperature and gestational age after adjustment for maternal and perinatal factors, air pollutants, and meteorological exposure. The associations between ambient minimum temperature and gestational age. (PDF 115 kb)

Additional file 3: **Figure S2.** The associations between ambient maximum temperature and gestational age after adjustment for maternal and perinatal factors, air pollutants, and meteorological exposure. The associations between ambient maximum temperature and gestational age. (PDF 106 kb)

Additional file 4: **Figure S3.** The associations between ambient maximum temperature and gestational age after adjustment for maternal and perinatal factors, air pollutants, and meteorological exposure. The associations between ambient maximum temperature and gestational age. (PDF 109 kb)

Additional file 5: **Figure S4.** The associations between ambient maximum temperature and birth weight after adjustment for maternal and perinatal factors, air pollutants, and meteorological exposure. The associations between ambient maximum temperature and birth weight. (PDF 82 kb)

Additional file 6: **Table S2.** The description of full-term birth and preterm birth by different ambient temperature category in Brisbane, 2000–2010 (*n* = 237,585). The description of full-term birth and preterm birth. (DOCX 23 kb)

Additional file 7: **Table S3.** The associations of ambient temperature at conception/delivery with preterm birth in Brisbane, 2000–2010 (*n* = 237,585). Association of ambient temperature with preterm birth. (DOCX 31 kb)

## Abbreviations

NO_2_: Nitrogen dioxide
O_3_: Ozone
PDCU: The Perinatal Data Collection Unit
PHA: The Public Health Act
PM_10_: Particulate matter with a diameter < 10 μm
SAS: The Statistical Analysis System
SO_2_: Sulfur dioxide
