# Supplementary material for: Exploring associations of maternal exposure to ambient temperature with duration of gestation and birth weight: a prospective study
Source: BMC Pregnancy Childbirth. 2018 Dec 29;18:513. doi: 10.1186/s12884-018-2100-y (PMC6311008; doi:10.1186/s12884-018-2100-y)
Supplement: Supplementary file 1 — Table S1. Summary distribution of meteorological and pollutant exposure of 237,585 births in Brisbane, 2000–2010. Summary distribution of meteorological and pollutant exposure. (DOCX 27 kb) [file 12884_2018_2100_MOESM1_ESM.docx]

Additional file 1: Table S1. Summary distribution of meteorological and pollutant exposure of 237,585 births in Brisbane, 2000-2010

|  |  | **Mean (SD)** |  | **Percentiles** | | | | | | | | | | | | | |
| --- | --- | --- | --- | --- | --- | --- | --- | --- | --- | --- | --- | --- | --- | --- | --- | --- | --- |
|  |  |  |  | **Min** |  | **5th** |  | **25th** |  | **50th** |  | **75th** |  | | **95th** | | **Max** |
| **Meteorological indicators** | | | | | | | | | | | | | | | | | |
| **In the first week** |  |  |  |  |  |  |  |  |  |  |  |  | |  | |  |  |
| Maximum temperature,℃ |  | 26.05 (3.26) |  | 18.41 |  | 20.70 |  | 23.19 |  | 26.54 |  | 28.66 | |  | | 30.80 | 33.39 |
| Minimum temperature,℃ |  | 15.75 (4.44) |  | 4.7 |  | 8.57 |  | 11.99 |  | 16.35 |  | 19.57 | |  | | 22.04 | 23.98 |
| Relative humidity, % |  | 99.94 (8.00) |  | 34.52 |  | 54.13 |  | 61.37 |  | 67.47 |  | 72.40 | |  | | 79.77 | 90.23 |
| Air pressure, Kpa |  | 1018.09 (4.39) |  | 1006.54 |  | 1010.30 |  | 1015.04 |  | 1018.13 |  | 1021.39 | |  | | 1024.80 | 1029.79 |
| **In the first four weeks** |  |  |  |  |  |  |  |  |  |  |  |  | |  | |  |  |
| Maximum temperature |  | 26.05 (3.06) |  | 19.68 |  | 21.14 |  | 23.16 |  | 26.81 |  | 28.65 | |  | | 30.40 | 31.68 |
| Minimum temperature |  | 15.76 (4.24) |  | 6.87 |  | 9.10 |  | 11.90 |  | 16.28 |  | 19.70 | |  | | 21.58 | 22.55 |
| Relative humidity |  | 66.98 (5.50) |  | 49.05 |  | 58.25 |  | 62.88 |  | 67.51 |  | 70.94 | |  | | 75.46 | 79.96 |
| Air pressure |  | 1018.08 (3.55) |  | 1009.75 |  | 1011.82 |  | 1015.73 |  | 1018.31 |  | 1020.89 | |  | | 1023.30 | 1025.21 |
| **In the last week** |  |  |  |  |  |  |  |  |  |  |  |  | |  | |  |  |
| Maximum temperature |  | 25.96 (3.28) |  | 18.41 |  | 20.61 |  | 23.09 |  | 26.46 |  | 28.63 | |  | | 30.80 | 33.39 |
| Minimum temperature |  | 15.80 (4.37) |  | 4.70 |  | 8.71 |  | 12.12 |  | 16.26 |  | 19.51 | |  | | 22.04 | 23.99 |
| Relative humidity |  | 67.22 (7.95) |  | 34.53 |  | 54.26 |  | 61.60 |  | 67.86 |  | 72.56 | |  | | 79.93 | 90.23 |
| Air pressure |  | 1018.14 (4.40) |  | 1006.54 |  | 1010.29 |  | 1015.06 |  | 1018.21 |  | 1021.39 | |  | | 1024.80 | 1029.73 |
| **In the last four weeks** |  |  |  |  |  |  |  |  |  |  |  |  | |  | |  |  |
| Maximum temperature |  | 25.96 (3.08) |  | 19.68 |  | 21.03 |  | 23.12 |  | 26.67 |  | 28.36 | |  | | 30.40 | 31.68 |
| Minimum temperature |  | 15.79 (4.15) |  | 6.87 |  | 9.23 |  | 12.14 |  | 16.18 |  | 19.58 | |  | | 21.58 | 22.55 |
| Relative humidity |  | 67.22 (5.42) |  | 49.05 |  | 58.46 |  | 63.13 |  | 67.65 |  | 71.13 | |  | | 75.63 | 79.96 |
| Air pressure |  | 780.43 (3.40) |  | 770.35 |  | 774.81 |  | 777.96 |  | 780.26 |  | 782.84 | |  | | 786.23 | 788.64 |
| **Air pollutants** |  |  |  |  |  |  |  |  |  |  |  |  | |  | |  |  |
| **In the first week** |  |  |  |  |  |  |  |  |  |  |  |  | |  | |  |  |
| PM_10_, μg/m^3^ |  | 16.45 (11.12) |  | 8.03 |  | 10.41 |  | 12.67 |  | 14.96 |  | 17.53 | |  | | 25.04 | 215.50 |
| O_3,_ ppm |  | 13.21 (3.77) |  | 3.45 |  | 7.77 |  | 10.64 |  | 12.67 |  | 15.36 | |  | | 20.14 | 28.11 |
| NO_2,_ ppm |  | 7.17 (3.49) |  | 1.46 |  | 2.71 |  | 4.37 |  | 6.23 |  | 9.84 | |  | | 13.86 | 17.31 |
| SO_2,_ ppm |  | 0.71 (0.48) |  | 0.00 |  | 0.01 |  | 0.27 |  | 0.73 |  | 1.07 | |  | | 1.44 | 2.17 |
| **In the first four weeks** |  |  |  |  |  |  |  |  |  |  |  |  | |  | |  |  |
| PM_10_ |  | 16.46 (6.61) |  | 10.60 |  | 11.83 |  | 13.70 |  | 15.32 |  | 17.31 | |  | | 22.84 | 79.40 |
| O_3_ |  | 13.20 (3.39) |  | 7.08 |  | 8.49 |  | 10.82 |  | 12.68 |  | 15.11 | |  | | 20.04 | 24.88 |
| NO_2_ |  | 7.16 (3.26) |  | 2.10 |  | 2.98 |  | 4.34 |  | 6.53 |  | 10.35 | |  | | 12.62 | 15.95 |
| SO_2_ |  | 0.71 (0.42) |  | 0.01 |  | 0.10 |  | 0.30 |  | 0.75 |  | 1.10 | |  | | 1.32 | 1.58 |
| **In the last week** |  |  |  |  |  |  |  |  |  |  |  |  | |  | |  |  |
| PM_10_ |  | 16.24 (6.48) |  | 8.03 |  | 10.10 |  | 12.49 |  | 14.73 |  | 17.23 | |  | | 25.04 | 215.50 |
| O_3_ |  | 13.06 (3.78) |  | 3.46 |  | 7.63 |  | 10.54 |  | 12.51 |  | 15.07 | |  | | 20.10 | 28.11 |
| NO_2_ |  | 7.11 (3.50) |  | 1.46 |  | 2.70 |  | 4.26 |  | 6.23 |  | 9.83 | |  | | 13.79 | 17.31 |
| SO_2_ |  | 0.74 (0.47) |  | 0.00 |  | 0.01 |  | 0.30 |  | 0.79 |  | 1.10 | |  | | 1.44 | 2.17 |
| **In the last four weeks** |  |  |  |  |  |  |  |  |  |  |  |  | |  | |  |  |
| PM_10_ |  | 16.21 (6.50) |  | 10.60 |  | 11.41 |  | 13.46 |  | 15.07 |  | 17.11 | |  | | 22.61 | 79.40 |
| O_3_ |  | 13.07 (3.41) |  | 7.08 |  | 8.48 |  | 10.61 |  | 12.56 |  | 15.11 | |  | | 19.75 | 24.88 |
| NO_2_ |  | 7.11 (3.23) |  | 2.10 |  | 2.96 |  | 4.33 |  | 6.39 |  | 10.19 | |  | | 12.61 | 15.95 |
| SO_2_ |  | 0.73 (0.41) |  | 0.01 |  | 0.10 |  | 0.32 |  | 0.78 |  | 1.11 | |  | | 1.32 | 1.58 |

**Abbreviations:** PM_10:_ particular matter with a diameter less than 10μm; O_3_: ozone; NO_2_: nitrogen dioxide; SO_2_: sulfur dioxide.
