# Supplementary material for: Exploring associations of maternal exposure to ambient temperature with duration of gestation and birth weight: a prospective study
Source: BMC Pregnancy Childbirth. 2018 Dec 29;18:513. doi: 10.1186/s12884-018-2100-y (PMC6311008; doi:10.1186/s12884-018-2100-y)
Supplement: Supplementary file 2 — Figure S1. The associations between ambient minimum temperature and gestational age after adjustment for maternal and perinatal factors, air pollutants, and meteorological exposure. The associations between ambient minimum temperature and gestational age. (PDF 115 kb) [file 12884_2018_2100_MOESM2_ESM.pdf]

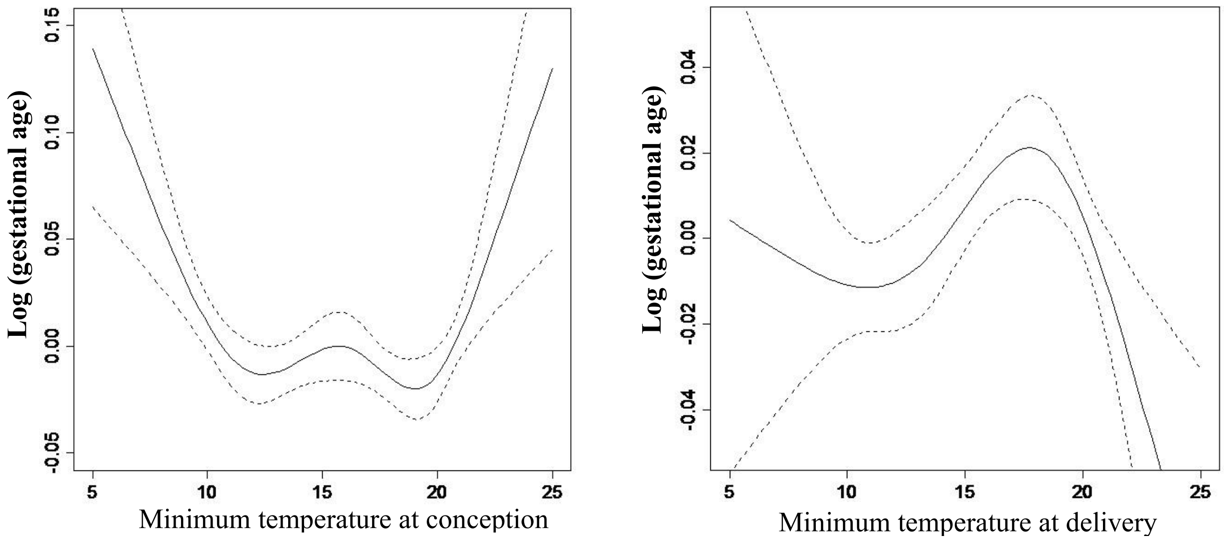

Online Figure 1. The associations between ambient minimum temperature and gestational age after adjustment for maternal and perinatal factors, air pollutants, and meteorological exposure

Minimum temperature at conception using average data of the first four weeks of gestation

Minimum temperature at delivery using average data of the last four weeks of gestation
