# Supplementary material for: Exploring associations of maternal exposure to ambient temperature with duration of gestation and birth weight: a prospective study
Source: BMC Pregnancy Childbirth. 2018 Dec 29;18:513. doi: 10.1186/s12884-018-2100-y (PMC6311008; doi:10.1186/s12884-018-2100-y)
Supplement: Supplementary file 3 — Figure S2. The associations between ambient maximum temperature and gestational age after adjustment for maternal and perinatal factors, air pollutants, and meteorological exposure. The associations between ambient maximum temperature and gestational age. (PDF 106 kb) [file 12884_2018_2100_MOESM3_ESM.pdf]

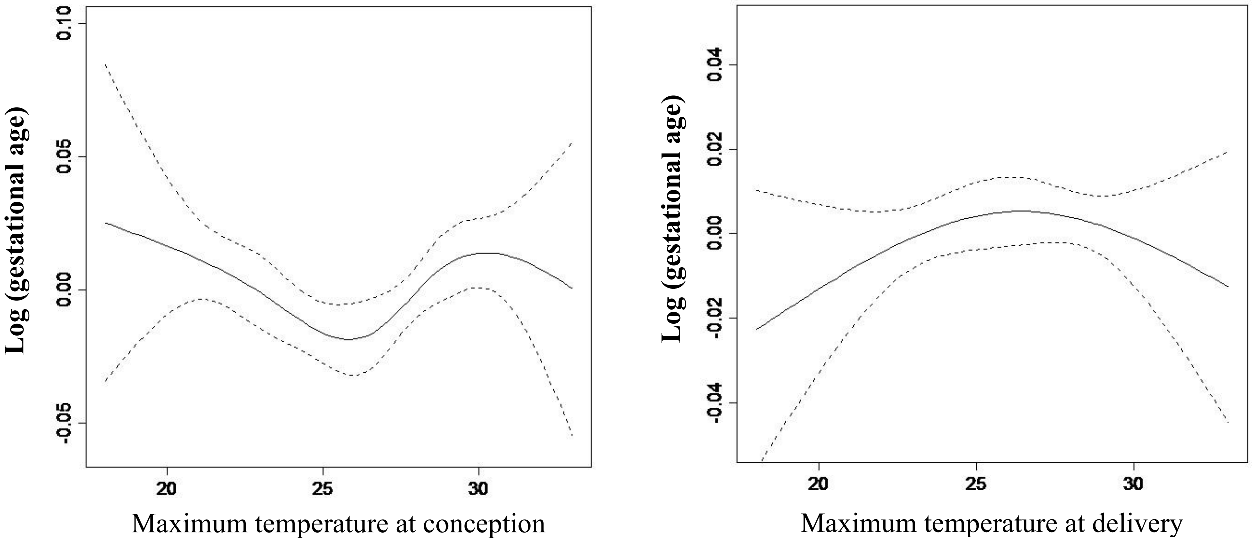

Online Figure 2. The associations between ambient maximum temperature and gestational age after adjustment for maternal and perinatal factors, air pollutants, and meteorological exposure

Maximum temperature at conception using data of the first week of gestation

Maximum temperature at delivery using data of the last week of gestation
