# Supplementary material for: Exploring associations of maternal exposure to ambient temperature with duration of gestation and birth weight: a prospective study
Source: BMC Pregnancy Childbirth. 2018 Dec 29;18:513. doi: 10.1186/s12884-018-2100-y (PMC6311008; doi:10.1186/s12884-018-2100-y)
Supplement: Supplementary file 5 — Figure S4. The associations between ambient maximum temperature and birth weight after adjustment for maternal and perinatal factors, air pollutants, and meteorological exposure. The associations between ambient maximum temperature and birth weight. (PDF 82 kb) [file 12884_2018_2100_MOESM5_ESM.pdf]

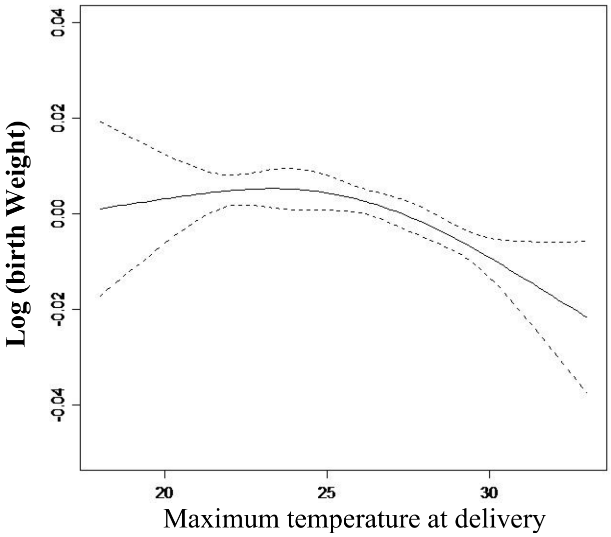

Online Figure 4. The associations between ambient maximum temperature at delivery and birth weight after adjustment for maternal and perinatal factors, air pollutants, and meteorological exposure

Maximum temperature at delivery using average data of the last four weeks of gestation
