# Supplementary material for: Exploring associations of maternal exposure to ambient temperature with duration of gestation and birth weight: a prospective study
Source: BMC Pregnancy Childbirth. 2018 Dec 29;18:513. doi: 10.1186/s12884-018-2100-y (PMC6311008; doi:10.1186/s12884-018-2100-y)
Supplement: Supplementary file 6 — Table S2. The description of full-term birth and preterm birth by different ambient temperature category in Brisbane, 2000–2010 (n = 237,585). The description of full-term birth and preterm birth. (DOCX 23 kb) [file 12884_2018_2100_MOESM6_ESM.docx]

**Additional file 6: Table S2. The description of full-term birth and preterm birth by different ambient temperature category in Brisbane, 2000-2010 (n=237,585)**

|  |  | **Full-term birth**  **(n)** | **Preterm bith**  **(n)** | **Proportion of**  **preterm birth (%)** | **χ^2^** | ***P*** |
| --- | --- | --- | --- | --- | --- | --- |
| **In the first week** |  |  |  |  |  |  |
| Maximum temperature |  |  |  |  | 2.031 | 0.566 |
| >30 |  | 25036 | 1688 | 6.32 |  |  |
| 25-30 |  | 114457 | 7724 | 6.32 |  |  |
| 20-25 |  | 77416 | 5280 | 6.38 |  |  |
| ≤20 |  | 5629 | 365 | 6.09 |  |  |
| Minimum temperature |  |  |  |  | 9.720 | **0.021** |
| >20 |  | 48701 | 3150 | 6.08 |  |  |
| 15-20 |  | 79646 | 5471 | 6.45 |  |  |
| 10-15 |  | 65957 | 4555 | 6.46 |  |  |
| ≤10 |  | 28234 | 1871 | 6.21 |  |  |
| **In the first four weeks** |  |  |  |  |  |  |
| Maximum temperature |  |  |  |  | 0.378 | 0.945 |
| >30 |  | 20091 | 1343 | 6.27 |  |  |
| 25-30 |  | 123720 | 8379 | 6.34 |  |  |
| 20-25 |  | 77196 | 5226 | 6.34 |  |  |
| ≤20 |  | 1531 | 103 | 6.30 |  |  |
| Minimum temperature |  |  |  |  | 22.79 | **<.001** |
| >20 |  | 49191 | 3182 | 6.08 |  |  |
| 15-20 |  | 78402 | 5419 | 6.46 |  |  |
| 10-15 |  | 70553 | 4927 | 6.53 |  |  |
| ≤10 |  | 24392 | 1519 | 5.86 |  |  |
| **In the last week** |  |  |  |  |  |  |
| Maximum temperature |  |  |  |  | 6.630 | 0.085 |
| >30 |  | 24043 | 1673 | 6.51 |  |  |
| 25-30 |  | 113486 | 7525 | 6.22 |  |  |
| 20-25 |  | 79035 | 5418 | 6.42 |  |  |
| ≤20 |  | 5974 | 431 | 6.73 |  |  |
| Minimum temperature |  |  |  |  | 10.365 | **0.016** |
| >20 |  | 47425 | 3312 | 6.53 |  |  |
| 15-20 |  | 81431 | 5317 | 6.13 |  |  |
| 10-15 |  | 67479 | 4626 | 6.42 |  |  |
| ≤10 |  | 26203 | 1792 | 6.40 |  |  |
| **In the last four weeks** |  |  |  |  |  |  |
| Maximum temperature |  |  |  |  | 3.343 | 0.342 |
| >30 |  | 20263 | 1397 | 6.45 |  |  |
| 25-30 |  | 120683 | 8057 | 6.26 |  |  |
| 20-25 |  | 80108 | 5483 | 6.41 |  |  |
| ≤20 |  | 1484 | 110 | 6.90 |  |  |
| Minimum temperature |  |  |  |  | 10.425 | 0.015 |
| >20 |  | 47776 | 3328 | 6.51 |  |  |
| 15-20 |  | 80676 | 5265 | 6.13 |  |  |
| 10-15 |  | 73799 | 5047 | 6.40 |  |  |
| ≤10 |  | 20287 | 1407 | 6.49 |  |  |
