# Supplementary material for: Exploring associations of maternal exposure to ambient temperature with duration of gestation and birth weight: a prospective study
Source: BMC Pregnancy Childbirth. 2018 Dec 29;18:513. doi: 10.1186/s12884-018-2100-y (PMC6311008; doi:10.1186/s12884-018-2100-y)
Supplement: Supplementary file 7 — Table S3. The associations of ambient temperature at conception/delivery with preterm birth in Brisbane, 2000–2010 (n = 237,585). Association of ambient temperature with preterm birth. (DOCX 31 kb) [file 12884_2018_2100_MOESM7_ESM.docx]

**Additional file 7: Table S3. The associations of ambient temperature at conception/delivery with preterm birth in Brisbane, 2000-2010 (n=237,585)**

|  |  | **Preterm birth vs. full-term birth** | | | | | | | | | | |
| --- | --- | --- | --- | --- | --- | --- | --- | --- | --- | --- | --- | --- |
|  |  | Crude | |  | Adjusted | | | | | | | |
|  |  |  |  |  | Model 1 | |  | Model 2 | |  | Model 3^a/b^ | |
|  |  | OR (95% CI) | *P value* |  | OR (95% CI) | *P value* |  | OR (95% CI) | *P value* |  | OR (95% CI) | *P value* |
| **In the first week** |  |  |  |  |  |  |  |  |  |  |  |  |
| Maximum temperature |  |  |  |  |  |  |  |  |  |  |  |  |
| >30 |  | Ref |  |  | Ref |  |  | Ref |  |  | Ref |  |
| 25-30 |  | 1.000 (0.997, 1.003) | 0.974 |  | 1.000 (0.998, 1.003) | 0.824 |  | 1.000 (0.997, 1.003) | 0.885 |  | 0.999 (0.997, 1.003) | 0.702^a^ |
| 20-25 |  | 1.001 (0.997, 1.004) | 0.690 |  | 1.000 (0.996, 1.002) | 0.376 |  | 0.999 (0.995, 1.003) | 0.521 |  | 0.998 (0.994, 1.003) | 0.415^a^ |
| ≤20 |  | 0.996 (0.989, 1.003) | 0.270 |  | 0.996 (0.990, 1.002) | 0.195 |  | 0.996 (0.989, 1.003) | 0.251 |  | 0.996 (0.989, 1.003) | 0.263^a^ |
| Minimum temperature |  |  |  |  |  |  |  |  |  |  |  |  |
| >20 |  | 0.996 (0.994, 0.999) | **0.009** |  | 0.999 (0.996, 1.001) | 0.199 |  | 0.998 (0.996, 1.001) | 0.206 |  | 0.997 (0.994, 1.000) | 0.056^a^ |
| 15-20 |  | Ref |  |  | Ref |  |  | Ref |  |  | Ref |  |
| 10-15 |  | 1.000 (0.998, 1.003) | 0.795 |  | 1.000 (0.998, 1.001) | 0.355 |  | 1.001 (0.998, 1.004) | 0.610 |  | 1.002 (0.999, 1.005) | 0.242^a^ |
| ≤10 |  | 0.998 (0.995, 1.001) | 0.193 |  | 0.998 (0.995, 1.000) | 0.094 |  | 1.000 (0.995, 1.004) | 0.968 |  | 1.002 (0.998, 1.007) | 0.281^a^ |
| **In the first four weeks** |  |  |  |  |  |  |  |  |  |  |  |  |
| Maximum temperature |  |  |  |  |  |  |  |  |  |  |  |  |
| >30 |  | Ref |  |  | Ref |  |  | Ref |  |  | Ref |  |
| 25-30 |  | 1.001 (0.997, 1.004) | 0.667 |  | 1.000 (0.997, 1.003) | 0.919 |  | 1.000 (0.997, 1.004) | 0.949 |  | 1.000 (0.997, 1.004) | 0.983^a^ |
| 20-25 |  | 1.001 (0.997, 1.004) | 0.689 |  | 0.999 (0.995, 1.002) | 0.367 |  | 1.001 (0.996, 1.004) | 0.813 |  | 1.000 (0.995, 1.005) | 0.988^a^ |
| ≤20 |  | 0.998 (0.986, 1.010) | 0.759 |  | 0.998 (0.987, 1.009) | 0.718 |  | 0.999 (0.988, 1.011) | 0.938 |  | 1.000 (0.988, 1.011) | 0.997^a^ |
| Minimum temperature |  |  |  |  |  |  |  |  |  |  |  |  |
| >20 |  | 0.996 (0.993, 0.998) | **0.004** |  | 0.999 (0.996, 1.001) | 0.235 |  | 0.996 (0.993, 0.999) | **0.042** |  | 0.996 (0.993, 0.999) | **0.039**^a^ |
| 15-20 |  | Ref |  |  | Ref |  |  | Ref |  |  | Ref |  |
| 10-15 |  | 1.001 (0.998, 1.003) | 0.609 |  | 1.001 (0.998, 1.003) | 0.606 |  | 1.001 (0.997, 1.003) | 0.725 |  | 1.002 (0.998, 1.005) | 0.383^a^ |
| ≤10 |  | 0.994 (0.991, 0.997) | **<.001** |  | 0.994 (0.991, 0.997) | **<.001** |  | 0.995 (0.991, 1.000) | 0.061 |  | 0.998 (0.992, 1.003) | 0.415^a^ |
| **In the last week** |  |  |  |  |  |  |  |  |  |  |  |  |
| Maximum temperature |  |  |  |  |  |  |  |  |  |  |  |  |
| >30 |  | Ref |  |  | Ref |  |  | Ref |  |  | Ref |  |
| 25-30 |  | 0.997 (0.994, 1.000) | 0.086 |  | 1.000 (0.997, 1.003) | 0.972 |  | 0.999 (0.996, 1.002) | 0.666 |  | 0.999 (0.996, 1.002) | 0.578^b^ |
| 20-25 |  | 0.999 (0.996, 1.003) | 0.603 |  | 1.002 (0.999, 1.005) | 0.126 |  | 1.000 (0.996, 1.004) | 0.981 |  | 1.000 (0.996, 1.003) | 0.891^b^ |
| ≤20 |  | 1.002 (0.996, 1.009) | 0.511 |  | 1.007 (1.002, 1.013) | **0.013** |  | 1.005 (0.998, 1.012) | 0.202 |  | 1.004 (0.999, 1.009) | 0.107^b^ |
| Minimum temperature |  |  |  |  |  |  |  |  |  |  |  |  |
| >20 |  | 1.004 (1.001, 1.007) | **0.003** |  | 1.002 (1.000, 1.004) | 0.125 |  | 1.003 (1.001, 1.006) | **0.026** |  | 1.004 (1.001, 1.007) | **<.006**^b^ |
| 15-20 |  | Ref |  |  | Ref |  |  | Ref |  |  | Ref |  |
| 10-15 |  | 1.003 (1.001, 1.005) | **0.020** |  | 1.003 (1.001, 1.005) | **0.004** |  | 1.000 (1.000, 1.005) | 0.075 |  | 1.000 (0.997, 1.003) | 0.981^b^ |
| ≤10 |  | 1.003 (0.999, 1.006) | 0.104 |  | 1.004 (1.001, 1.008) | **0.009** |  | 1.000 (0.995, 1.005) | 0.978 |  | 1.000 (0.995, 1.005) | 0.932^b^ |
| **In the last four weeks** |  |  |  |  |  |  |  |  |  |  |  |  |
| Maximum temperature |  |  |  |  |  |  |  |  |  |  |  |  |
| >30 |  | Ref |  |  | Ref |  |  | Ref |  |  | Ref |  |
| 25-30 |  | 0.998 (0.995, 1.002) | 0.285 |  | 0.999 (0.996, 1.002) | 0.642 |  | 0.998 (0.995, 1.002) | 0.327 |  | 0.998 (0.995, 1.002) | 0.340^b^ |
| 20-25 |  | 0.999 (0.996, 1.003) | 0.814 |  | 1.002 (0.999, 1.006) | 0.154 |  | 1.000 (0.995, 1.004) | 0.828 |  | 0.999 (0.994, 1.004) | 0.681^b^ |
| ≤20 |  | 1.005 (0.992, 1.017) | 0.475 |  | 1.005 (0.995, 1.016) | 0.332 |  | 1.002 (0.992, 1.014) | 0.710 |  | 1.005 (0.993, 1.017) | 0.453^b^ |
| Minimum temperature |  |  |  |  |  |  |  |  |  |  |  |  |
| >20 |  | 1.004 (1.001, 1.007) | **0.005** |  | 1.002 (1.000, 1.006) | 0.052 |  | 1.003 (1.001, 1.007) | **0.048** |  | 1.004 (1.001, 1.007) | **0.022**^b^ |
| 15-20 |  | Ref |  |  | Ref |  |  | Ref |  |  | Ref |  |
| 10-15 |  | 1.003 (1.001, 1.005) | **0.022** |  | 1.003 (1.001, 1.005) | **0.002** |  | 1.001 (0.998, 1.005) | 0.509 |  | 1.001 (0.098, 1.004) | 0.976^b^ |
| ≤10 |  | 1.004 (1.000, 1.006) | **0.052** |  | 1.004 (1.001, 1.008) | **0.007** |  | 1.003 (1.001, 1.006) | **0.016** |  | 1.002 (1.000, 1.005) | **0.047^b^** |

Model 1: adjusted for maternal age at delivery, indigenous status, maternal marital status, mode of labor onset, parity, baby’s gender, and birth weight.

Model 2: further adjusted for relative humidity and air pressure around conception and delivery, along with all air pollutants (PM_10,_ O_3,_ NO_2,_ and SO_2_) around conception and delivery.

Model 3^a^: model 2 adjustment plus calendar month at delivery, as well as maximum temperature and minimum temperature around delivery.

Model 3^b^: model 2 adjustment plus calendar month at delivery, as well as maximum temperature and minimum temperature around conception.
